# Native and Engineered Probiotics: Promising Agents against Related Systemic and Intestinal Diseases

**DOI:** 10.3390/ijms23020594

**Published:** 2022-01-06

**Authors:** Haokun Shen, Zitong Zhao, Zengjue Zhao, Yuyi Chen, Linghua Zhang

**Affiliations:** Guangdong Provincial Key Laboratory of Protein Function and Regulation in Agricultural Organisms, College of Life Sciences, South China Agricultural University, Guangzhou 510642, China; haokunshen@stu.scau.edu.cn (H.S.); zitong@stu.scau.edu.cn (Z.Z.); zj_zhao@stu.scau.edu.cn (Z.Z.); chenyuyi122004@foxmail.com (Y.C.)

**Keywords:** *Escherichia coli* Nissle 1917, *Akkermansia muciniphila*, *Clostridium butyricum*, lactic acid bacteria, *Bifidobacterium* spp., inflammatory bowel disease, cancer, diabetes, obesity

## Abstract

Intestinal homeostasis is a dynamic balance involving the interaction between the host intestinal mucosa, immune barrier, intestinal microecology, nutrients, and metabolites. Once homeostasis is out of balance, it will increase the risk of intestinal diseases and is also closely associated with some systemic diseases. Probiotics (*Escherichia coli* Nissle 1917, *Akkermansia muciniphila*, *Clostridium butyricum*, lactic acid bacteria and *Bifidobacterium* spp.), maintaining the gut homeostasis through direct interaction with the intestine, can also exist as a specific agent to prevent, alleviate, or cure intestinal-related diseases. With genetic engineering technology advancing, probiotics can also show targeted therapeutic properties. The aims of this review are to summarize the roles of potential native and engineered probiotics in oncology, inflammatory bowel disease, and obesity, discussing the therapeutic applications of these probiotics.

## 1. Introduction

The benefits of probiotics are well known, and both traditional and recently discovered probiotics have received a lot of attention. They can also be quite successful therapeutic agents. There is a large number of reviews that focus on describing single probiotics [1,2,3], their derivatives [4,5], or probiotics in terms of health/disease [6,7]. The variety of probiotic bacteria and the complex relationships between them and various diseases cannot be summarized in a single review. We aim to enumerate and discuss the beneficial effects of probiotics (*Escherichia coli* Nissle 1917, *Akkermansia muciniphila*, *Clostridium butyricum*, lactic acid bacteria and *Bifidobacterium* spp.) and, once intestinal homeostasis is compromised, their impacts on cancer, inflammatory bowel disease (IBD), obesity, and other systemic disorders. Moreover, we will elaborate on the benefits of their modified bacteria (including their derivatives). In this review, comprehensive elucidation of these probiotics, both native and modified, is expected to have a significant impact on a deeper understanding of probiotics.

## 2. Intestinal Probiotics

*Escherichia coli* Nissle 1917 (EcN) was initially isolated from the feces of a German soldier who was clear of intestinal disease in a *Shigella* spp. heavily contaminated area [8]. Since its discovery, it has been commonly used to regulate intestinal microbiota [9,10], relieve inflammation [11,12], etc. Furthermore, EcN has good colonization [13,14], is nonimmunogenic, and has a clear genetic background [15], making it easily genetically engineered for therapeutic use. 

*Akkermansia muciniphila* (AM), a strictly anaerobic, non-motile, non-spore-forming, intestinal mucin-degrading, Gram-negative bacteria, was isolated in 2004 by Derrien et al. [16]. Recently, the EFSA Panel on NDA concluded that the novel food (NF), pasteurized AM, is safe for the target population at 3.4 × 10^10^ cells/day, provided that the number of viable AM is below 10 cells/g NF [17]. AM is a resident probiotic with a high abundance in the mucus layer of the intestine [18,19]. It is thought to modulate host immune homeostasis, improve metabolism, protect the intestinal barrier [20], show anti-aging and anti-cancer effects [21], and play a role in the microbiota–brain–gut axis [22]. AM is currently being extensively researched, and expected to be the next generation of probiotics.

*Clostridium butyricum* (CB) is a strictly anaerobic, butyric acid-producing, Gram-positive bacteria, colonizing predominantly the distal small intestine and colon [23]. CB can be used as a probiotic preparation to promote host health, including regulation of intestinal microbiota [24], production of beneficial metabolites [25,26], prevention of intestinal inflammation [27,28], promotion of growth [29,30], etc. Another major advantage of the CB application is that it is a bacillus that forms heat-resistant endospores and survives in adverse conditions [31]. 

Over the past few decades, lactic acid bacteria and *Bifidobacterium* spp. have been used in dairy fermentation around the world [32,33]. Lactic acid bacteria is an aerotolerant anaerobic Gram-positive bacteria, which usually colonies the mucosal surface of the intestine [34] in the function of maintaining the balance of intestinal microbiota [35,36] and improving immune function [37,38]. Similarly, *Bifidobacterium* spp. is a Gram-positive bacterium, which has a variety of probiotic functions. It is able to adhere to the epithelial cells of the intestine [39] to secrete antibacterial substances [40] and synthesize vitamins [41]. In addition, they both have a hydrolytic effect on bile, which can cause oxidative stress and cellular damage [42]. Their tremendous worth in the fields of food and medicine has been demonstrated through many years of research.

The intestinal microbiota maintains the balance of the intestinal micro-ecology and links to almost all digestive diseases. Digestive disorders are the underlying basis for many systemic diseases, including endocrine diseases. Increased intestinal permeability and abnormal nutrient absorption contribute to these diseases, and further affect a range of metabolic changes. Probiotics, when used correctly, are a crucial part of maintaining human health.

## 3. Native Probiotics and Diseases 

### 3.1. Native Probiotics and Cancer 

**Figure 1:**
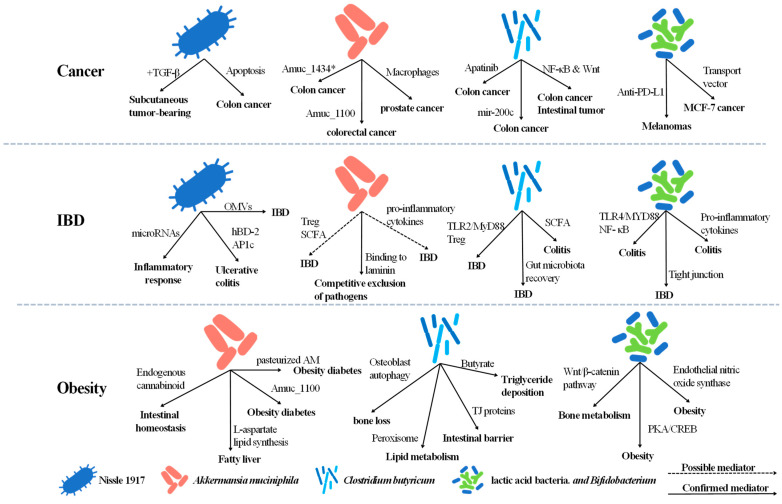
Effects of native probiotics in diseases. *Escherichia coli* Nissle 1917, *Akkermansia muciniphila*, *Clostridium butyricum*, lactic acid bacteria, and *Bifidobacterium* spp. have been proven to be promising therapeutic agents in cancer, IBD, and obesity.

Cancer is a major public health problem worldwide. Traditional cancer treatments include surgery, radiation therapy, and chemotherapy, which often lead to serious side effects as well as the destruction of adjacent normal cells [43]. Intestinal carcinogenesis is closely associated with alterations in intestinal native microbiota [44]. This may suggest that some probiotic bacteria can be utilized to assist in cancer prevention or therapy (Figure 1). 

#### 3.1.1. Native *Escherichia coli* Nissle 1917 (EcN) and Cancer

EcN can selectively colonize and replicate within solid tumors [45]. Except for tumor colonization, EcN can also trigger apoptosis in colon cancer HT-29 cells through a dose- and time-dependent mechanism [46]. In subcutaneous tumor-bearing mice, combined treatment of EcN and TGF-β blockers has been shown to be superior to monotherapy, and this synergy is probably mediated by the interaction of tumor-specific effector T cells, antigen-specific IFN-γ^+^ CD8^+^ T cells, and the gut microbiota [47]. However, 1917 can also produce colibactin in vitro and in vivo, inducing DNA damage, which may contribute to the development of colorectal cancer [48].

#### 3.1.2. Native *Akkermansia muciniphila* (AM) and Cancer

AM is closely associated with cancer. It is found to be more abundant in non-small cell lung cancer patients than normal healthy people [49], and the abundance of AM gradually decreases during the progression of cirrhosis only to hepatocellular carcinoma-cirrhosis [50]. AM in combination with current anti-tumor therapies, including cisplatin treatment and IL-2-based immunotherapy, is also a promising treatment strategy [51,52]. In addition, the drug treatments of castration-resistant prostate cancer [53], pancreatic ductal adenocarcinoma [54], lung cancer [55], and colorectal cancer [56,57] may also be driven by AM.

AM has some specific proteins that may impact the progression of tumors. Among these, the Amuc_1434* degrades mucin 2 in colon cancer cells and promotes apoptosis of human colon cancer LS174T cells [58,59], and its specific outer membrane protein Amuc_1100 can inhibit colitis-associated colorectal cancer by regulating CD8^+^ cytotoxic T lymphocytes [60].

AM also plays an essential role in immunization. AM has been reported to suppress colorectal tumorigenesis and prostate cancer by inducing tumor-associated macrophages [61,62]. Notably, AM may contribute to immune checkpoint blockade (ICB). AM is a responder during anti-PD-1 immunotherapy and has an impact on effectiveness [63,64,65], perhaps due to the alterations in glycerophospholipid metabolism [66] and AM-derived cdAMP [67]. 

#### 3.1.3. Native *Clostridium butyricum* (CB) and Cancer

CB reduces colitis-related colon cancer [68] and intestinal tumor [69] in mice, which may be related to the regulation of the NF-κB, Wnt signaling pathway. In addition, CB and its related metabolites also play a role in regulating the gut microbiota and promoting cancer therapy. CB achieved 18% eradication rates of *Helicobacter pylori* infection (associated with gastric cancer) among outpatients in a pilot study [70]. The cell-free supernatants derived from CB possess an antibiofilm effect and inhibit the growth of Enterotoxigenic *Bacteroides fragilis* [71], which has a risk of causing colorectal cancer. Since CB belongs to bacillus, the orally spores encapsulated by prebiotics have also been reported to specifically enrich and suppress colon cancer, regulating the intestinal microbiota [72]. It is notable that CB also influences microRNAs; it promotes the upregulation of tumor suppressor mir-200c, leading to the regulation of colitis-induced oncogenesis [73].

CB can also be used as an adjunct to current therapies. The research of Tian et al. has shown that CB reduces adverse events, especially diarrhea, caused by chemotherapy for lung cancer [74]. Furthermore, CB exhibits a synergistic effect when combined with lapatinib treatment for colon tumors in mice [75]. In ICB therapy, CB therapy can enhance the efficacy even in antibiotic-treated patients and is associated with longer progression-free survival and overall survival [76].

#### 3.1.4. Native *Bifidobacterium* spp., Lactic Acid Bacteria and Cancer

*Bifidobacterium* spp. and lactic acid bacteria have been widely used in food, healthcare and medical applications. As an important component of intestinal probiotics, they also have an important contribution to anti-cancer. Chou et al. showed that dietary *Limosilactobacillus fermentum* appears to modulate the intestinal microbiota and reduce inflammation, which may well be helpful in mitigating the development of colon cancer [77], and the exopolysaccharides of lactic acid bacteria have the capability to induce apoptosis in cancer cells [78]. In addition, solid fermented grains of *Limosilactobacillus reuteri* and *Lactiplantibacillus plantarum* subsp. *plantarum* are also anti-proliferative against cancer cells in vitro, which may be related to released bioactive peptides and degraded polysaccharides [79].

A decrease in lactic acid bacteria and *Bifidobacterium* spp. was observed in patients with colorectal cancer [80]. In addition, enteral administration of *Bifidobacterium breve* can usually maintain the total fecal organic acids of patients with malignancies on chemotherapy above 100 μmol/g, with a pH below 7.0, and reduce the frequency of fever and antibiotic use (compared to the placebo group) [81]. This all suggests that we can intervene in the development of cancer through probiotics. Currently, lactic acid bacteria have been used as a vector to successfully transport therapeutic agents into MCF-7 cancer cells in vitro [82]. However, the safety and efficacy of this drug vector in vivo have yet to be explored. On the other hand, encouragingly, the significant independent antitumor effects and adjuvant effect on anti-PD-L1 of live *Bifidobacterium* spp. have been demonstrated in mice melanomas, which may be related to an anti-tumor T-cell response [83]. This bodes well for the use of *Bifidobacterium* spp. in human immunotherapies.

### 3.2. Native Probiotics and Inflammatory Bowel Disease (IBD)

IBD is a chronic recurrent inflammation of the gastrointestinal tract, mainly including Crohn’s disease and ulcerative colitis (UC) [84]. At present, more and more novel and emerging therapies towards IBD are put into research and development [85]. A general consensus has been established that gut microbiota play pivotal roles in the development of IBD [86]. Probiotics and their produced metabolites are crucial for gut homeostasis, suggesting they may have a role in the treatment of IBD [87,88] (Figure 1).

#### 3.2.1. Native *Escherichia coli* Nissle 1917 (EcN) and IBD

EcN has been assessed in clinical trials and has shown a remarkable therapeutic effect, especially in UC treatment [89]. As early as 2004, EcN was shown to have the same effects as regular mesalazine but required a considerably lower dosage to reduce UC symptoms. [90]. EcN was also reported to protect intestinal epithelial cells by inducing human beta-defensin-2, which showed a higher expression level in UC than in CD, through NF-κB and activator protein 1 [91]. Meanwhile, EcN is a gut-friendly microorganism. Specific pathogen-free, but not germ-free, IL-2^−/−^ mice generally develop colitis. Strikingly, GF IL-2^−/−^ mice with EcN colonized did not induce colitis [92]. This is consistent with EcN being more effective in treating UC in the clinic. Remarkably, EcN was also shown to affect the regulation of micro-RNAs involved in the inflammatory response (miR-143, miR-150, miR-155, miR-223, and miR-375). For example, EcN could reverse the downregulation of miR-150, which reduced the expression of c-Myb and thereby compromised the integrity of the intestinal barrier [93,94]. It could also suppress the up-regulation of miR-155 and miR-233, which is consistent with the activities of IL-1β expression and the Th1/Th17 response, respectively [95]. 

EcN can also assist in the performance of other drugs. Common drugs for the treatment of IBD, such as 5-ASA and mesalamine, may affect the degradation rates of the polysaccharide-based drug delivery system. Fortunately, the utilization rate of oral drugs has been shown to be improved under the assistance of probiotics including EcN [96]. Additionally, EcN outer membrane vesicles (OMVs) have also been proved to have intestinal barrier protective and anti-inflammatory effects towards IBD, and it could be a promising treatment because of its non-replication and safety [91,97].

#### 3.2.2. Native *Akkermansia muciniphila* (AM) and IBD

As an anaerobic bacteria specialized in the utilization of mucin as a carbon and nitrogen source, AM became a key force to protect the integrity of the epithelial barrier and update the mucus layer, which plays a key role in the onset of IBD [98,99,100]. The abundance of AM can be markedly enriched, along with restoring the epithelium barriers in acute colitis, by hyaluronic acid-bilirubin nanomedicine [101]. Additionally, AM could firmly bind to the laminin and cultured colonic epithelial cell lines Caco-2 and HT-29, thus gaining competitive exclusion of pathogens [102]. Plenty of investigations on IBD patients have identified that the abundance of AM is critically influenced. Through obtaining colonic biopsies and mucus brushings from UC patients, Earley et al. found that lower abundances of AM had a strong association with a lower percentage of sulphated mucin and higher inflammation [103]. Furthermore, 53.7% of IBD patients who underwent washed microbiota transplantation had a clinical response while also showing a higher colonization rate of AM [104]. Strikingly, different strains of AM could perform special functions on immune modulation. Both strain 139 and strain ATCC BAA-835 were sufficient to downregulate the expression of the pro-inflammatory cytokines (TNF-α and IFN-γ), while ATCC BAA-835 could modulate the differentiation of regulatory T (Treg) cells and increase the production of short-chain fatty acids, but strain 139 could not [105]. This suggests that more research needs to be done to better apply AM in IBD treatment.

#### 3.2.3. Native *Clostridium butyricum* (CB) and IBD

Generally, microbial dysbiosis in IBD patients is associated with a decrease in short-chain fatty acid (SCFA)-producing bacteria [106,107]. CB can consume undigested dietary fiber and generate SCFAs, especially butyrate and acetate, and is listed as a potential probiotic for treating IBD [1]. Accordingly, the derivatives of CB have been shown to effectively prevent bloody diarrhea, therefore treating dextran sulfate sodium (DSS)-induced experimental colitis in rats [108]. It has also been reported that antibiotics used early in life play an important role in gut microbiota disorders, and can lead to IBD, while supplementation of antibiotics with butyrate can restore the intestinal macrophage response and prevent T cell dysfunction [109]. Compared with mesalamine and sodium butyrate, CB CGMCC0313 performed better on lowering the serum levels of both IL-23 and TNF-α as well as appearing to restore the intestinal microbiota more quickly [110]. CB CBM588 was shown to directly trigger the TLR2/MyD88 pathway of intestinal macrophages in the mucosa, resulting in the generation of IL-10, which can suppress colitis by regulating Treg cells [111,112]. In addition, CB can also be combined with other prebiotics or immunotherapy to assist in the treatment of IBD. For instance, CB capsules combined with specific immunotherapy can significantly inhibit UC and down-regulate the expression of proinflammatory cytokines in the clinics [113]. CB also employs germinated barley foodstuff, which exerts a therapeutic effect on IBD as a fermentation substrate. Combining them, according to this, CB can effectively treat colitis [114].

#### 3.2.4. Native *Bifidobacterium* spp., Lactic acid Bacteria and IBD

*Bifidobacterium* spp. and Lactic acid bacteria are probiotics that have been widely applied in the food and medicine industry [115]. Plenty of plant extracts in the treatment of IBD showed that the reduction of colitis symptoms in mice was accompanied by a significant increase in *Bifidobacterium* spp. and Lactic acid bacteria, such as purple sweet potato anthocyanin extract, glycerol monolaurate, polysaccharides from tea flowers, etc. [116,117,118]. The underlying mechanism of action is closely related to the activation of Toll-like receptors and their downstream signaling pathways [119]. On the one hand, it has been shown that the TLR4/MYD88/NF- κB signal pathway can be inhibited by *Bifidobacterium animalis* subsp. *lactis* XLTG11, causing downregulation of pro-inflammatory cytokines to alleviate DSS-induced colitis in mice [120]. On the other hand, the mice can also be protected by *Bifidobacterium adolescentis* IF1-03, which activated macrophages through the TLR2/ERK/p38 MAPK signal pathway and induced differential regulation of the inflammatory immune response in the Treg/Th17 axis [121]. Mediated by the same signal pathway, it has been found that strain *Bifidobacterium bifidum* 1 could also induce significant and continuous enhancement of the intestinal tight junction (TJ) barrier in Caco-2 monolayers [122]. In IBD mediated by pathogenic bacteria, they have been proven to regulate the tight junction of Caco-2 cells [123], counteract Adherent-Invasive *Escherichia coli* virulence in CD patients, and reduce the secretion of cytokines related to the IL-23/Th17 axis in UC patients [124]. Furthermore, secreted factors from *Bifidobacterium dentium* can also down-regulate the endoplasmic reticulum stress gene and regulate the unfolded protein response to promote MUC2 secretion [125].

### 3.3. Native Probiotics and Obesity with Associated Diseases

Obesity is prone to a number of complications and is particularly associated with the development of diabetes [126]. The main reason for the development of obesity is a high-fat diet (HFD). HFD can imbalance the gut microbiota, reducing some probiotic bacteria such as *Bifidobacterium*, *Lactobacillus* and *Akkermansia* [127]. Moreover, it damages the intestinal barrier and even leads to a leaky gut, resulting in a range of metabolic diseases [128]. On the other hand, consumption of probiotics can improve the integrity of the mucosal barrier [129]. Furthermore, the intestinal microbiota has a regulatory role in obesity, with probiotics having a potentially beneficial effect on various metabolic parameters [130,131] (Figure 1). 

#### 3.3.1. Native *Escherichia coli* Nissle 1917 (EcN) and Obesity with Associated Diseases

To our knowledge, there are no reports related to the direct effects of native EcN on obesity and diabetes. However, EcN increases the expression of tight junction proteins to stabilize the intestinal barrier, which may be related to its secretion of OMVs and soluble factors [132,133]. One of the mechanisms is through the inhibition of TNF-*α* and IFN-*γ*-induced activation of NF-κB p65, thereby decreasing the binding of NF-κB in the myosin light-chain kinase promoter to reduce the disruption of tight junctions [134]. In addition, during this process, some miRNAs (miR-203, miR-483-3p, miR-595) may also contribute to tight junctions [135]. Subsequently, EcN was shown to have a protective effect against enteropathogenic *E. coli*-induced intestinal epithelial barrier dysfunction [136] and 5-fluorouracil-induced epithelial cell damage [137]. Considering the protective effect of EcN against pathogen-induced and chemical-induced intestinal barrier damage, it may also mitigate the disruption of the intestinal barrier caused by HFD to hinder the development of other metabolic diseases. 

#### 3.3.2. Native *Akkermansia muciniphila* (AM) and Obesity with Associated Diseases

It is now generally accepted that the abundance of AM is negatively correlated with obesity [138,139,140]. Dietary habits, one of the main causes of obesity, can also change the abundance of AM [141]. Dietary intervention in mice on an HFD can increase the abundance of AM [142]. Interestingly, a large amount of research has shown that while using some substances (including grape polyphenols [143], epigallocatechin-3-gallate [144], rhubarb extract [145], polysaccharides obtained from *Cordyceps militaris* [146], protein-bound β-glucan [147], puerarin [148], Bofutsushosan [149,150], etc.) to combat obesity, it has also been found that they can regulate the abundance of AM to improve efficacy. On the other hand, low AM abundance is associated with type 2 diabetes [139,141] and leads to impaired insulin secretion and disturbed glucose homeostasis even in lean individuals [151]. Therefore, it is natural that research on AM is focusing on the fight against obesity and its related diseases, especially diabetes. 

The anti-obesity properties of AM have been almost comprehensively evaluated. It improves vital obesity parameters and insulin sensitivity, and rescues glucose homeostasis in HFD mice [152]. Moreover, AM treatment can increase intestinal endogenous cannabinoid levels to further maintain intestinal homeostasis [139]. Clinical studies have also shown that the higher the AM abundance, the healthier the insulin sensitivity in individuals with calorie-restricted weight loss [153]. Meanwhile, AM-metabolites have been reported to affect various transcription factors and to be involved in peripheral lipid metabolism [154]. In addition to systemic obesity, AM has shown excellent performance in improving fatty liver and liver function through the enterohepatic axis, possibly in part by increasing *L*-aspartate levels from the intestine to the liver and regulating genes involved in lipid synthesis [152,155,156]. AM also makes a significant contribution to the intestinal barrier. There is evidence that AM helps to repair the intestinal barrier [152], and contrarily AM deficiency damages the intestinal barrier integrity [157]. This maintenance of the intestinal barrier may be related to the inflammation-relieving and anti-cancer properties of its membrane protein, Amuc_1100 [60]. Interacting with Toll-like receptor 2, Amuc_1100 also improves metabolism in obese and diabetic mice [158]. Combined with the reducing perilipin 2 (associated with lipid storage) in brown and white adipose tissues as well as increased energy expenditure and excretion by pasteurized AM [159], we can conclude that the beneficial effects of pasteurized AM on obesity and diabetes still exist. 

#### 3.3.3. Native *Clostridium butyricum* (CB) and Obesity with Associated Diseases

The tendency to accumulate body fat is negatively correlated with the abundance of CB and an HFD will lead to a reduction in the amount of CB [160]. In turn, CB administration reduced adipose tissue mass, blood glucose, and serum triglycerides [161,162,163], increased peroxisome abundance, and modulated related genes to improve lipid metabolism [161]. Additionally, CB can also mitigate leaky gut and reinforce the intestinal barrier by upregulating TJ proteins (claudin-1 and occludin) [162]. The cell wall components and secretory products of CB (butyrate) have also been observed to stimulate ANGPTL4 production, which affects the triglyceride deposition in adipocytes [164]. 

On the other hand, CB has a beneficial effect on the postoperative period of bariatric-related surgery. A comparison of the modified Gastrointestinal Quality of Life Index before and after the intervention shows that CB improves gastrointestinal symptoms after gastric bypass surgery, which may be caused by a combination of altered digestive environment and microbiota [165]. In addition, CB has an interventional effect on bone metabolism. Studies have shown that administration of CB can promote osteoblast autophagy to reduce bone loss after bariatric surgery, possibly also by altering the microbial composition of the gut [166]. 

Moreover, CB improves glucose homeostasis and insulin resistance in type 2 diabetic mice [167]. In autoimmune diabetes, CB also has a protective effect, which involves the regulation of intestinal immunity, particularly pancreatic regulatory T-cells and intestinal microbiota [168]. Through the brain–gut axis, CB was shown to attenuate cerebral I/R injury with diabetes and to reverse neuronal apoptosis [169]. In conclusion, CB has a promising application in improving obesity, diabetes, and other diseases, as well as helping patients to recover after surgery.

#### 3.3.4. Native *Bifidobacterium* spp., Lactic Acid Bacteria and Obesity with Associated Diseases

Significant differences in microbial composition were found in the childhood obesity survey, with a significant decrease in the abundance of *Bifidobacterium* [170]. Unsurprisingly, a relatively high abundance of *Lacticaseibacillus paracasei* subsp. *paracasei* has an effect against obesity in children [171]. Adjusting the abundance of probiotics can help prevent obesity in the early stages. *Bifidobacterium animalis* subsp. *lactis* CECT 8145 treatment inhibited intake and significantly increased plasma adiponectin levels in obese mice, which may play a key role in the regulation of insulin sensitivity and glucose metabolism [172]. *Bifidobacterium animalis* subsp. *lactis* A6 reduces body weight and fat mass in obese mice, even showing better results than AM [173]. It increases the expression of endothelial nitric oxide synthase which alters mitochondrial metabolism and reduces the risk of obesity [173]. Intervention with *Lactiplantibacillus plantarum* subsp. *plantarum* reduces the rate of weight gain in mice through the peroxisome proliferators-activated receptor pathway, improves related parameters, attenuates obesity-related oxidative damage and inflammatory responses [174]. Similarly, *Bifidobacterium longum* [175], *Bifidobacterium pseudolongum* [176], *Lactiplantibacillus plantarum* subsp. *plantarum* [177], *Lacticaseibacillus rhamnosus* [178], *Companilactobacillus crustorum* [178], *Latilactobacillus sakei* subsp. *sakei* [179,180], and *Lactobacillus johnsonii* [181] all induce weight loss, shown by reduced fat accumulation in HFD-induced obese mice. Notably, via the gut–bone axis, *Bifidobacterium pseudocatenulatum* mitigates the adverse effects of obesity on bone metabolism; one of the reasons for this may be through activation of the Wnt/β-catenin pathway [182].

In addition, *Bifidobacterium longum* contributes to glucose tolerance, regulates lipid metabolism, protects the liver and pancreas, and improves glucagon-like peptide-1 (GLP-1) [183]. After inactivation, it still has the potential to improve obesity and diabetes-related parameters [184]. *Lacticaseibacillus paracasei* subsp. *paracasei* was also reported to have an ameliorative effect on rats with type 2 diabetes mellitus, even with parameters comparable to those of normal mice after the intervention [185]. In clinical studies, *Limosilactobacillus reuteri* has been reported to improve insulin sensitivity in some patients [186]. It has good therapeutic effects in patients with type 2 diabetes and can increase the abundance of *Bifidobacterium* [187]. *Lactobacillus acidophilus* improves the expression of genes related to glucose and lipid metabolism and regulates the gut microbiota to combat diabetes [188]. Meanwhile, it also enhances the intestinal barrier function [188]. Likewise, *Bifidobacterium* has a modulating function on intestinal permeability and has a promising application in intestinal leakage, but with attention to the antagonistic effects when combined with prebiotics [189]. 

The combination of *Lacticaseibacillus rhamnosus* and *Bifidobacterium lactis* showed a protective effect on islet B cell function in children with newly diagnosed type 1 diabetes [190]. The culture supernatant of *Bifidobacterium longum* and *Lacticaseibacillus rhamnosus* promotes the “browning” of adipose tissue, altering energy metabolism and activating thermogenesis through PKA/CREB to treat obesity [191]. The mechanism of the combination of *Bifidobacterium* spp. and Lactic acid bacteria in the treatment of obesity still remains to be explored and the appropriate form of microbiota supplementation needs to be screened.

### 3.4. Summary

Currently, the anti-tumor effects of probiotics have mainly been reported in digestive system cancer and lung cancer. Probiotic therapies used in oncology are mainly investigated in cells and mice. This cancer treatment is mostly used as an adjuvant, with mechanisms of probiotics that modulate the composition of the gut microbia and promote immunological responses. Although probiotic therapy is still in its infancy, it can be added to the daily diet for cancer prevention. In addition, probiotics can also be used in combination with drugs and ICB in clinical treatment.

The research on probiotic therapy in IBD treatment is mainly carried out on clinical patients or experimental mouse models, especially colitis. The main responsibilities of probiotics are intestinal microbiota regulation, intestinal epithelial barrier protection, and abnormal inflammatory response inhibition. Existing probiotic therapy can be combined with prebiotics or immunotherapy to achieve a significant therapeutic effect; some of them even catch up with the general clinical drugs.

Obesity is closely linked to gut microbes. One of the mechanisms of using probiotics to treat obesity lies in the regulation of the intestinal microbiota composition. The microbiota affects glucose and lipid metabolism, obesity-related biochemical parameters, inflammation, and oxidative stress levels, insulin sensitivity, and the intestinal barrier through various signaling pathways. When the impacts are negative, a series of metabolic disturbances and pathological changes can lead to the development of obesity. The utilization of probiotics as an appropriate supplement is expected to reverse this process, counteracting the development of obesity and its complications.

Native probiotics have a wide range of applications and are beneficial to health. They show a preventive and palliative effect on many diseases. 

## 4. Modified Probiotics and Diseases

It is possible to modify the properties of probiotics in a targeted way, even transforming its auxiliary role in disease treatment into a mainstay. Purposefully engineered probiotics can be more multi-functional and efficient. 

### 4.1. Modified Escherichia coli Nissle 1917, EcN

**Figure 2:**
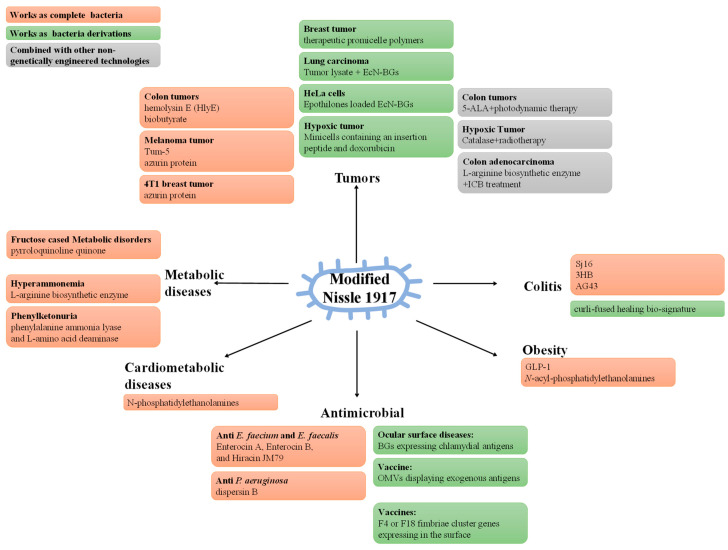
Strategies of modified EcN in diseases. There are three main strategies for modified EcN: 1. Expressing direct therapeutic factors. 2. Using the expressed therapeutic factors to complement other therapies. 3. Using EcN proper or its derivatives as carriers, relying on targeting or immune activity to transport the therapeutic factors.

The complete genomic DNA sequence has given a strong impetus to the engineering methods of EcN [15]. As an engineered bacterium, EcN is expected to achieve better pharmacological properties. In recent years, the engineered EcN has been studied in a variety of fields (Figure 2) (Table 1).

#### 4.1.1. Express Direct Therapeutic Factors

One of the key strategies for using the engineered EcN in disease therapy is to modify it to directly express therapeutic factors. The administration of engineered HlyE-expressing EcN in cytolytic therapy of tumors promotes regression of tumor tissues in mice [192]. In this research, EcN was equipped with a strictly regulated expression system. This process includes extinguishing the *araFGH* operon and *araBAD* operon, and knocking down *ptsG* to ensure persistent inducibility of L-arabinose, which induces P*_BAD_*, thereby expressing HlyE. In addition, engineered EcN expressing soluble Tum-5 (a fragment of tumstatin) [193] or azurin, a copper-containing redox protein that can initiate cancer cell apoptosis [194], effectively inhibits tumor growth in xenograft mice tumor cells. Similarly, the tumor-targeting characteristics allow the introduction of a cluster of biosynthetic genes for cytotoxic compounds into EcN for tumor therapy [45,195]. 

In terms of intestinal inflammation, EcN is used in the expression of *schistosome* immunoregulatory proteins to continuously protect the body against colitis [196]. In this study, the sequence encoding Sj16 peptide was linked to the *HlyA* C-terminal signaling sequence and contained in an expression vector alongside the *HlyB* and *HlyD*. Using HlyB, HlyD, and TolC proteins to form the pore recognizing HlyA protein signal, Sj16 was secreted directly into the extracellular environment, rescuing gut microbial compositions and effectively alleviating DSS-induced colitis in mice. The heterologous synthesis of (R)-3-hydroxybutyrate (3HB) by altering the EcN metabolic pathway has also been studied [197]. This process involved screening for acetyl-CoA acetyltransferase, 3HB-CoA dehydrogenase, and thioesterase isozymes, inserting the optimum 3HB synthesis system into the EcN genome, and knocking out *adhE* or *ldhA* to inhibit the branch pathways and increase 3HB production, thereby alleviating colitis. Optogenetics has also been combined with probiotics, using upconversion microgels to convert near-infrared light into local visible blue light [198]. Through this technology, the recombinant EcN is activated to secrete autotransporter 43 adhesins antigen, achieving precise spatiotemporal regulation, which effectively alleviates colitis in mice [198]. Recently, recombinant IL-10-secreting EcN has been used in the optotheranostic nanosystem for UC as a real-time intervention module for long-lasting relief of the intestinal inflammatory responses [199], making it possible to diagnose and treat intestinal inflammation at home.

In particular, to combat obesity and prevent related chronic diseases, EcN has been modified to secrete dipeptidyl peptidase 4-degradation-resistant GLP-1 [200] or *N*-acyl-phosphatidylethanolamines. Both possess anti-obesity properties [201], which have numerous benefits for obese mice. These studies offer an oral treatment option for obesity and even chronic diseases.

The engineered EcN also has great potential in terms of antimicrobial activity. EcN was selected to deliver anti-enterococcal peptides, which were expressed as a fusion of Microcin V secretion tag and a mature bacteriocin [202]. These probiotics were subsequently demonstrated to reduce the levels of *Enterococcus faecium* and *Enterococcus faecalis* in the feces of male Balb/cJ mice. During intestinal infection with *Pseudomonas aeruginosa*, the EcN containing the ‘Sense-Kill’ construct could express dispersin B, which destabilizes biofilms against *P. aeruginosa* infection [203].

The engineered EcN has also been applied in cardiometabolic diseases. Producing N-phosphatidylethanolamines, endogenous anorexigenic lipids, EcN was constructed, which has been shown to reduce body weight, liver inflammation, fibrosis, and atherosclerotic necrosis, thus alleviating cardiovascular metabolic disease in low-density lipoprotein receptor null mice [204]. 

In metabolic diseases, EcN can still play a role. The modified probiotic EcN, producing pyrroloquinoline quinone (PQQ) and other metabolizing enzymes, is reported to relieve heavy metal toxicity [205], iron deficiency [206], fructose-induced dyslipidemia, hyperglycemia, and hepatic steatosis [206,207]. Moreover, to alleviate hyperammonemia, Kurtz et al. deleted the negative regulator of L-arginine biosynthesis and inserted a feedback-resistant L-arginine biosynthetic enzyme that exhausts ammonia for L-arginine biosynthesis in EcN, which yielded satisfactory results in a Phase I clinical study [208]. Modification of EcN is also an alternative strategy to fight against phenylketonuria (PKU). The genes encoding phenylalanine ammonia-lyase and L-amino acid deaminase are inserted into the genome to facilitate EcN consumption of phenylalanine in patients with PKU [209]. These findings all demonstrated the potential of EcN to be used in the treatment of metabolic disorders.

#### 4.1.2. Express Adjuvant Therapeutic Factors

Another alternative strategy is to express adjuvant therapeutic factors. Engineered EcN is also used in combination therapy with photodynamic therapy. It was tailored by metabolic engineering to promote 5-aminolevulinic acid (5-ALA) production, which can be converted to protoporphyrin IX (the photosensitizer for photodynamic therapy) [210]. In particular, *Salmonella arizona hemA*^M^ and endogenous *hemL* were co-expressed in EcN to increase 5-ALA accumulation. Meanwhile, levulinic acid was used to inhibit downstream biosynthesis for 5-ALA production. Moreover, to improve the efficacy of radiotherapy, a continuous catalase (CAT)-secreting EcN has been constructed, using CAT to catalyze the production of O_2_ from H_2_O_2_ in tumor cells, alleviating the hypoxic environment and increasing the sensitivity of tumors to radiotherapy [211]. 

Engineered EcN also serves for ICB treatment. Considering the enhancement on T-cell anti-tumor activity of L-arginine in tumors [226], similarly to Kurtz et al. [208], EcN has been engineered to maintain a high local level of L-arginine in tumors, which was achieved by deleting the arginine repressor gene (*ArgR*) and integrating feedback-resistant mutant *N*-acetyl glutamate synthase *Arg^Afbr^* [212]. Therefore, PD-L1 blockade therapy can be coordinated with this probiotic, which utilizes ammonia, metabolic waste in tumors, to produce L-arginine. 

Another application idea is to use the quorum sensing of bacteria. For example, cholera autoinducer 1 expressing EcN greatly reduced the lethality of *Vibrio cholerae* through communication [213,214]. 

#### 4.1.3. EcN or EcN-derivatives as a Targeted Delivery System of Therapeutic Factors

The engineered EcN can also be employed as a delivery system of therapeutic factors. Xie et al. used acid-labile materials that are hydrolyzed responsively to the acidic tumor microenvironment to conjugate therapeutic promicelle polymers on EcN [215,216]. The polymers are released and self-assembled into hybrid micelles that are endocytosed into tumor cells in response to cytosolic GSH and release synergetic anti-tumor drugs [215], achieving a tumor-targeted release of drugs in vivo. Considering that this drug loading is friendly to bacterial motion profiles and activity and that the drug accumulation is higher than that of commonly used nanocarriers [216], this technology offers an effective strategy for targeted delivery. 

Interestingly, Joshi et al. constructed an EcN that secretes a curli-fused healing bio-signature, where these curli fibers bind firmly to the inflammation site of the intestinal mucus layer like a band-aid [217], and they systematically designed the pMUT plasmid containing the curli secretion system to export curli proteins with specific therapeutic factors to the intestine in vivo [218]. This study could be a boon for patients with IBD and even intestinal trauma. 

As a new adjuvant and antigen delivery platform, the bacterial ghosts (BGs) based on EcN to treat cancer have also been studied in recent years. Kraśko et al. showed that a course of three subcutaneous inoculations with Lewis lung carcinoma (LLC) lysate supplemented with EcN-BGs significantly increased the survival rate of mice after removal of LLC tumors [219]. Effective drug delivery is also a major challenge in drug therapy, and thus cell-targeted drug delivery vehicles are receiving a lot of attention in oncology drug therapy. EcN is reported to effectively load Epothilones and induce apoptosis in HeLa cells [220]. Recently, Xie et al. used the photothermal effect of EcN-BGs surface-modified nanorods to modulate the temporal and spatial release of contained drugs [221], which suggests that it is a promising candidate for drug carrier. Except for cancer, EcN-derived BGs expressing chlamydial antigens have been constructed, providing a new insight into the treatment of ocular surface diseases [222].

Based on the OMVs lacking infectivity while retaining pathogen-associated molecular pattern molecules and the specific regulation of T cells via TLR2 [223,227], the display of exogenous antigens on the surface of EcN OMVs using a ClyA fusion chimera to trigger cellular immune responses has also proven to be a great strategy for the development of recombinant subunit vaccines [223].

EcN may be used in the development of live vaccines. Porcine postweaning diarrhea is closely associated with F4 and F18 fimbriae enterotoxigenic *Escherichia coli* (ETEC), and studies have shown that EcN integrated with F4 or F18 fimbriae cluster genes expressed in the surface can activate adhesion inhibition of F4^+^ and F18^+^ ETEC by piglets [224]. This evidence suggests that EcN is a promising candidate for a live vaccine.

Interestingly, in addition to regular-sized forms, the nanosized forms of EcN have been studied in the production of tumor-targeting delivery. By deleting the *minCD* gene and enhancing the expression of the *minE* gene, EcN-derived minicells were produced on the scale [225]. Compared with traditional nanoparticles, these minicells have higher biocompatibility, lower immunogenicity and toxicity, greater drug loading and therapeutic index [228], providing an innovative means of drug delivery. The EcN-derived minicells harboring a low-pH insertion peptide (to increase molecular accumulation in the acidic tumor microenvironment) and doxorubicin (antibiotic antineoplastics) show a significant tumor regression ability in a mouse model [225].

### 4.2. Modified Akkermansia muciniphila

AM was isolated in 2004 [16], and in 2011 its genome was sequenced and annotated by Smidt et al. [229], laying the foundation for genetic modification of AM. However, to our knowledge, there are no reports of AM genetic modification. Apart from the probiotic effect of AM, studies have focused on its OMVs (Table 2). AM-OMVs can influence obesity-related genes, as well as metabolism, to treat obesity, improve the tight junction to control leaky gut [230,231,232], and decrease the level of pro-inflammatory cytokines to ameliorate colitis [233], maintaining intestinal homeostasis. Subsequently, AM-OMVs may alleviate pathological damage to the liver and prevent liver fibrosis via the hepatic portal vein [234,235]. Not limited to the intestine axis, though the gut–brain axis, AM-derived OMVs can also affect the expression of key genes in the serotonin system and promote serotonin concentrations [236,237]. In addition, AM-OMVs exhibit significant osteogenic effects and inhibit osteoclast differentiation, conveying the potential to regulate bone homeostasis [238]. Moreover, AM-OMVs elevate the proportion of GZMB^+^ and IFN-γ^+^ lymphocytes in CD8^+^ T cells and recruit macrophages, which represent their antitumor potential [62]. 

Based on their powerful and broad therapeutic properties, AM-OMVs may be designed for drug delivery in the future. For example, through engineering, AM-OMVs can act on the surface as immunotherapeutic agents. In fact, it is not only OMVs; given the probiotic effect of AM (Section 3.1.2, Section 3.2.2 and Section 3.3.2), its ability to colonize, and the clear genetic background, AM could be genetically modified using λ-Red recombination system/CRISPR-Cas system/nisin-controlled gene expression system to display or secrete the therapeutic factors for application in more fields to treat diseases.

### 4.3. Modified Clostridium Butyricum

Currently, CB is hardly ever engineered for health maintenance and disease prevention (Table 2), while it is mainly applied in biosynthesis [262,263,264]. Most of these studies involve modifying metabolic pathways to optimize production through gene overexpression or inactivation. From a health perspective, our laboratory constructed a porcine epidermal growth factor overexpressing CB to enhance its gut protective function [240]. However, it should be mentioned that the major hindrance to the application of engineered CB in health care is the paucity of viable genetic tools for CB, or in other words, genetic manipulation of CB is prone to failure. 

In general, for gene editing in CB, a plasmid system for shuttling and replication in *E. coli* and CB is first required. A number of plasmids have been successfully applied to *Clostridium* spp. including plasmids such as pMTL007 [263,265] and pMTL82151 [240,266]. In terms of gene editing methods, Heap et al. developed the ClosTron system in 2007 [267]. The principle of this technique is to use type II intron RNA to self-splice into lariat RNAs that complementarily pair with the target DNA sequence, thus achieving inactivation of the target gene [267]. Subsequently, this team developed another effective *Clostridium* spp. gene-editing technique, the allele-coupled exchange, which achieves gene knockout by bi-directional screening based on the *pyrE* gene [268]. Recently, efficient gene editing tools developed in CB using the heterologous Type II CRISPR-Cas9 system and endogenous Type I-B CRISPR-Cas system have also been reported [269]. This easily applied technology reduces the occurrence of the polarity effect in genetic manipulation for CB and lays the foundation for CB-based therapies.

In addition, CB-OMVs are highly productive and can stimulate the innate immune system [239], which suggests that CB-OMVs also have the potential to be used as vectors to deliver antigens or carry therapeutic factors by genetic manipulation.

### 4.4. Modified Lactic Acid Bacteria and Bifidobacterium spp.

#### 4.4.1. Treatment of Diseases

As traditional probiotics engineered Lactic acid bacteria and *Bifidobacterium* spp. have also been used in recent years (Table 2). *Bifidobacterium longum* was reported to be modified to express a single-chain variable fragment that bound to the receptor on the HER2-positive cancer cell surface [270] or Tum [271], thus inhibiting the growth and proliferation of cancer cells. 

Besides cancer, some studies also use *Bifidobacterium longum* to express antioxidant enzymes [241], anti-inflammatory cytokines [243], and hormones [242] against inflammatory diseases. Engineered *Lacticaseibacillus paracasei* subsp. *paracasei F19* can provide the enzyme *N*-acyl-phosphatidylethanolamine-preferring phospholipase D, which indirectly produces a natural lipid, palmitoylethanolamide, playing a role in regulating inflammation [250]. Moreover, through modulating the intestinal microbiota and reducing bacterial translocation to the liver, IL-22-expressing *Limosilactobacillus reuteri* has been reported to alleviate ethanol-induced steatohepatitis [247] and reduce nonalcoholic fatty liver diseases [246]. These constructed probiotics exert a powerful anti-inflammatory capacity, especially shown in the hepatic and intestinal axis.

Additionally, metabolic disorders are closely associated with the development of inflammation [272,273]. To alleviate metabolic disorders, *Bifidobacterium longum* has also been genetically engineered to express hormones originally released from the intestine (for example, oxyntomodulin and GLP-1) or its derivatives to facilitate the administration of drugs [244,245]. These engineered probiotics improve the absorption of nutrients, providing a new way to treat diabetes and obesity.

In fact, considering the brain–intestine axis, this may also be applied to the treatment of neurological disorders. Chen et al. constructed GLP-1-producing *Lactococcus lactis MG1363* and demonstrated its neuroprotective effects in Alzheimer’s disease mice and Parkinson’s disease mice, possibly through the TLR4/NF-κB and AKT/GSK3β signaling pathways [248]. Orally, angiotensin-expressing *Lacticaseibacillus paracasei* subsp. *paracasei* has also been found to promote beneficial circulating neurotransmitters and reduce neuroinflammatory cytokine expression in the prefrontal cortex [249]. Interestingly, the engineered Lactic acid bacteria in these studies both increased the abundance of AM, indicating that they may have a synergistic effect and can be considered in combination in treatment regimens.

#### 4.4.2. Probiotic Vaccines

Another crucial application for them is shown in probiotic vaccines (Table 2). Many studies have used probiotics to express the antigens of some pathogens and stimulate the body to secrete antibodies in large quantities. In pig farming, *Lacticaseibacillus casei* was modified to constitutively express E2 protein, the envelope protein of bovine viral diarrhea virus (BVDV) that possesses important epitopes, fused with cholera toxin B as an adjuvant to help antigen presentation [251]. Similarly, this strategy has also been applied to prevent the infection of porcine transmissible gastroenteritis virus (TGEV) and porcine epidemic diarrhea virus (PEDV) [252,253,254,259]. In fish culture, the recombinant *Lacticaseibacillus casei* expressing antigen VP2 of infectious pancreatic necrosis virus (IPNV) can induce a higher systemic immune response against infection to protect rainbow trout [255,256]. In addition to viruses, the presence of pathogenic bacteria can also cause economic damage to the pig industry. *Lacticaseibacillus casei* was chosen to express F4 fimbrial adhesin main subunit and conjunction with adjuvants to combat F4^+^ enterotoxigenic *Escherichia coli* infections [258]. The anti-bacteria effect of the engineered *Lacticaseibacillus casei* carrying antigens of the corresponding pathogenic bacteria was also demonstrated in mouse models of *Staphylococcus aureus* [260] and *Clostridium perfringens* [257] infection. In general, Lactic acid bacteria have the potential to become oral vaccines against infection. 

Another strategy is to use probiotics to express the receptor for the virus, thereby trapping the virus and reducing its ability to spread. *Lactobacillus acidophilus* was constructed by chromosomal integration to surface display human CD4 [261]. This engineered bacterium can capture HIV-1 and reduce the efficiency of HIV-1 transmission. However, this strategy may be difficult to intercept non-enterohepatic axis-invading pathogens.

### 4.5. Summary

Currently, the main strategy for the modification of probiotics is the expression of therapeutic factors for direct or adjuvant therapy. The others are to use them or their derivatives (BGs, OMVs, minicells, etc.) as carriers, utilizing their targeting or immunoactivities to transport therapeutic factors or to display antigens on the surface for probiotic vaccines. Probiotic treatment is mainly manifested in the intestinal tract and dispersed through the intestine–liver axis, the brain–intestinal axis, and so on. It has a greater potential for the treatment of digestive and metabolic diseases and some neurological disorders. Probiotic engineering has also excelled in the fight against infection via competition, quorum sensing, or masquerading itself as a host cell to hinder pathogens, but this often seems to be limited to the gut.

## 5. Challenges and Outlooks

As our friends, probiotics can bring positive health benefits and offer new treatments for diseases. However, the application of probiotic therapy still faces a range of risks and challenges.

The security of probiotics is an essential challenge for their applications. Notably, there is a pathogenicity island on the genome of EcN that produces colibactin, a genotoxin that induces DNA damage [48]. While much research has focused on its positive effects, we can never lose sight of this negative one. To develop EcN without colibactin risk, polyphosphate kinase inhibitors (e.g., mesalamine) can be used to inhibit colibactin production [274]. Another strategy is to delete or mutate virulence genes/virulence expression-dependent genes. However, we have to ascertain whether such manipulation will affect the normal metabolic function of the corresponding bacteria or not. 

Although AM currently appears to be a safe probiotic. There are still some contrary observations implying that AM is not a probiotic in all cases. For example, a high abundance of AM was detected in samples from patients with type 2 diabetes [275], and associated with the development of inflammation in some cases [276,277]. In addition, as a mucosal degrading bacterium, AM also disrupts mucus barrier function [278], which may lead to mucosal immune damage. These controversies may be due to various periods/stages of disease development or to different host states/microenvironments. For the maximum value of AM, it is now imperative to clarify its exact mechanism and how it interacts with the host and commensal bacteria, so we can prevent damage caused by its misuse.

CB has also been reported to acquire the type E botulinum neurotoxin gene [279,280]. Therefore, we should carefully select the engineered strains and manage them to avoid the acquisition of exogenous genes and to prevent the flow of their genes to other organisms. However, during the period of clinical treatment, isolated adverse cases have been reported where the administration of CB for biliary cancer has led to bacteremia [281]. This may be related to reduced immunity and a broken epithelial barrier, enlightening us to take into account the condition of patients to determine if probiotic therapy is appropriate. 

In contrast, Lactic acid bacteria and *Bifidobacterium* spp. appear to be relatively safe. They are commonly added as ingredients in food products and no serious adverse effects have been observed.

Additionally, there are risks associated with the use of modified bacteria. Exogenous genes can be transferred to other organisms through genetic drift, resulting in contamination of the gene pool in the ecosystem. Moreover, specific antibiotic resistance genes are often used as marker genes in the genetic manipulation of bacteria. This resistance gene has a certain probability of being transferred to other organisms, making them develop resistance. In modified probiotics application, attention also needs to be paid to the possibility of these bacteria triggering a violent immune response (due to the exogenous proteins) that could jeopardize the safety of the organism. It has to be admitted that every treatment involves a certain amount of risk. Some bacteria may have side-effects reported, but we cannot cover the whole picture with bias. Considering the positive impact of probiotics in the treatment of various diseases, we should not abandon the use of promising probiotics for isolated cases. Therefore, it is necessary to determine the population for which probiotics are suitable, including assessments of the age, gender, health level, and so on.

Common methods of administration include oral, injectable, and nebulized inhalation methods, while probiotic administration can be most conveniently done orally. Therefore, the ability of probiotics to adhere and colonize can often be critical to the efficacy of the treatment. Yang et al. found that EcN with defective adsorption to epithelial cells did not exhibit the original efficacy against tumors [47]. Meanwhile, adherence is the basis for colonization, and the capacity to adhere can also affect the dose of probiotics. Therefore, we should seek to improve the adherence of probiotics when applying them, for example, heterologous expressing surface proteins or cellular components that affect adhesion [282]. Following this, we still need to reassess the safety (paying particular attention to whether immunogenicity is altered) and efficacy of the engineered probiotics.

Currently, the mechanism of how probiotics achieve a protective effect on a particular organ is under-researched. Therefore, the strategy of using modifications to strengthen the original bacteria can be hampered. Furthermore, the interactions between multi-organs are still ambiguous, which limits the scope of probiotic application. Combined with the complexity of interactions among strains and the complex dietary factors [283], these difficulties make it hard for us to judge the role played by a specific probiotic in a particular microenvironment. In general, for the development of probiotic therapy, we need to strengthen the basic research related to probiotics, including the mechanism of the interactions between probiotics and different organs, the relationship between probiotics and commensal microorganisms, etc. 

## 6. Conclusions

Native probiotics can confer health on the host when applied in moderation. They can be effective in the treatment of cancer, IBD, obesity, and other related diseases. The mechanisms behind them mainly include the regulation of the composition of the microbiota in the microenvironment, the beneficial effects of secreted substances and their bacteriological components, and activation of the immune system on the host. 

Further modification of probiotics can give these native properties more targeted therapeutic factors that will deepen their efficacy and expand their application areas. However, the modification of probiotics is mainly based on the understanding of how probiotics play a probiotic role and how they interact with the organism/microenvironment. The existing inadequate theories of the underlying mechanisms may lead to unjustified modifications. Therefore, the safety of modified probiotics needs to be rigorously verified at the time of application.

## Figures and Tables

**Table 1:** The strategy of modified EcN in the treatment of diseases.

Strategy		Mechanisms	Functions/Benefits	Reference
Express direct therapeutic factors	express HlyE	the cytotoxicity of released HlyE	against tumors	[192]
	express Tum-5	the anti-angiogenesis effects of released Tum-5	against tumors	[193]
	express azurin	azurin selectively kills cancer cells	against tumors	[194]
	express colibactin	the cytotoxicity of released colibactin	against tumors	[45]
	express glidobactin	the cytotoxicity of released glidobactin	against tumors	[45]
	express luminmide	the cytotoxicity of released luminmide	against tumors	[45]
	express butyrate	the cytotoxicity of released butyrate	against tumors	[195]
	express Sj16	via Ruminococcaceae/butyrate/retinoic acid axis	against colitis	[196]
	express 3HB	regulate gut microbiota, relieve architectural changes, inflammatory cell infiltrations, and epithelial injuries	against colitis	[197]
	express autotransporter 43 adhesins antigen	use optogenetics to activate secretion	against colitis/precise spatiotemporal colonization	[198]
	express IL-10	phone visual diagnosis and optogenetics based secretion	against colitis/mobile health service	[199]
	express GLP-1 analog	GLP-1 analog diminishes food intake	against obesity	[200]
	express N-acyl-phosphatidylethanolamines	N-acyl-phosphatidylethanolamines induce satiety	against obesity	[201]
	express antimicrobial peptides	antimicrobial peptides target and kill *Enterococcus*	against vancomycin-resistant *Enterococcus*	[202]
	develop a synthetic genetic system	sense and kill *Pseudomonas aeruginosa*	against *Pseudomonas aeruginosa*	[203]
	express N-acyl-phosphatidylethanolamines	reduce body weight, liver inflammation, fibrosis and atherosclerotic necrosis	against cardiovascular metabolic disease	[204]
	express PQQ and other metabolizing enzymes	relieve oxidative stress	against heavy metal toxicity/iron deficiency/fructose-induced dyslipidemia/hyperglycemia/hepatic steatosis	[205,206,207]
	express l-arg biosynthetic enzyme	exhausts ammonia for L-arginine biosynthesis	against hyperammonemia	[208]
	express phenylalanine ammonia lyase and L-amino acid deaminase	consume phenylalanine within the gastrointestinal tract	against PKU	[209]
Express adjuvant therapeutic factors	express 5-ALA	5-ALA contributes to photodynamic therapy	against cancer	[210]
	express CAT	increase the production of O2 contributing to radiotherapy	against cancer	[211]
	produce high local concentrations of arginine	the enhancement on T-cell anti-tumor activity of L-arginine in tumors	against cancer	[212]
	express cholera autoinducer 1	use the qurom sensing of bacteria	against *Vibrio cholerae*	[213,214]
Targeted delivery system	connect therapeutic promicelle polymers on bacteria	connection material responsively to acidic tumor microenvironment to release drug	against cancer	[215,216]
	secrete curli-fused healing bio-signature	curli fibers bind firmly to inflammation site and transport therapeutic factors	against colitis	[217,218]
	use EcN-GBs as an adjuvant	induce cellular immune responses	against LLC	[219]
	EcN-GBs load Epothilones	the cytotoxicity of released Epothilones	against cancer	[220]
	modified EcN-GBs temporal and spatial release of contained drugs	using photothermal effect of nanorods to modulate release	release modulation	[221]
	express chlamydial antigens	induce cellular immune responses	against ocular surface diseases	[222]
	display exogenous antigens (ClyA fusion chimera) on the surface of EcN-OMVs	induce cellular immune responses	recombinant subunit vaccines deliver	[223]
	express F4 or F18 fimbriae in the surface of EcN	induce cellular immune responses	live vaccine application	[224]
	EcN-derived minicells loaded with a low-pH insertion peptide and doxorubicin	greater drug loading and therapeutic index contribute to doxorubicin tumor regression	against cancer	[225]

**Table 2:** The strategy of modified probiotics or probiotic derivatives in the treatment of diseases.

Probiotics		Strategy	Mechanisms	Functions/Benefits	Reference
*Akkermansia muciniphila*		OMVS delivery/treatment	regulate inflammation, energy homeostasis, intestinal barrier	against obesity	[230]
		OMVS delivery/treatment	activate the AMPK pathway and increase TJ gene expressions	against leaky gut	[231,232]
		OMVS delivery/treatment	regulate inflammation, epithelial stability	against DSS-induced colitis	[233]
		OMVS delivery/treatment	reduce inflammation, reverse the activation of hepatic stellate cells and normalize serum glucose, lipid profiles, liver enzymes	against HFD/CCl_4_-induced liver fibrosis	[234]
		OMVS delivery/treatment	promote osteogenesis and inhibit osteoclastogenesis	against osteoporosis	[238]
		OMVS delivery/treatment	regulate CD8^+^ T cells and macrophages	against prostate cancer	[62]
		OMVS delivery/treatment	induce serotonin biosynthesis	promote serotonin	[236]
*Clostridium butyricum*		OMVS delivery/treatment	enhance proinflammatory cytokine production	stimulate the immune system	[239]
		overexpress epidermal growth factor	activate STAT3 signal pathway and inhibit inflammation	gut protection	[240]
*Bifidobacterium longum*		express RhMnSOD	regulate inflammatory cytokines	against DSS-induced colitis	[241]
		express α-melanocyte-stimulating hormones	inhibit NF-κB p65 expression	against DSS-induced colitis	[242]
		express interleukin-12	upregulate the expression of Th1 cytokines (IFN-γ and TNF-α)	against coxsackie virus B3-induced myocarditis	[243]
		express oxyntomodulin	reduces food intake, body weight and blood lipid levels	against obesity	[244]
		express GLP-1	improve the efficiency of glucose control	against type 2 diabetes	[245]
Lactic acid bacteria	*Limosilactobacillus reuteri*	express IL-22	regulate REG3 via STAT3	against nonalcoholic fatty liver	[246]
	*Limosilactobacillus reuteri*	express IL-22	induce expression of REG3G	against ethanol-induced steatohepatitis	[247]
	*Lactococcus lactis*	express GLP-1	downregulate TLR4/NF-κB, upregulated the AKT/GSK3β signaling pathway and reverse disturbed microbiota	against Alzheimer/Parkinson	[248]
	*Lacticaseibacillus paracasei* subsp. paracasei	express angiotensin	increase beneficial circulating neurotransmitters and reduce neuro-inflammatory gene expression	benefits the gut-brain axis	[249]
	*Lacticaseibacillus paracasei* subsp. *paracasei*	express palmitoylethanolamide	block mucosal immune cell infiltration and the release of pro-inflammatory cytokines	against colitis	[250]
	*Lacticaseibacillus casei*	express bovine viral diarrhea virus E2 protein	induce cellular immune responses	against BVDV	[251]
	*Lacticaseibacillus casei*	express antigenic site of TGEV S protein and major antigen site of PEDV S protein	induce cellular immune responses	against TGEV and PEDV	[252,253,254]
	*Lacticaseibacillus casei*	express IPNV protein antigen VP2	induce cellular immune responses	against IPNV	[255,256]
	*Lacticaseibacillus casei*	express toxoid of *Clostridium perfringens* α-toxin	induce cellular immune responses	against *Clostridium perfringens*	[257]
	*Lacticaseibacillus casei*	express the F4 fimbrial adhesin main subunit	induce cellular immune responses	against F4+ enterotoxigenic *Escherichia coli*	[258]
	*Lactiplantibacillus plantarum* subsp. *plantarum*	express PEDV S1 protein	induce cellular immune responses	against PEDV	[259]
	*Lactiplantibacillus plantarum* subsp. *plantarum*	express *Staphylococcus aureus* nontoxic mutated α-hemolysins	induce cellular immune responses	against *Staphylococcus aureus*	[260]
	*Lactobacillus acidophilus*	express Human CD_4_ on the surface	capture and neutralize HIV-1	against HIV-1	[261]

## Data Availability

No new data were created or analyzed in this study. Data sharing is not applicable to this article.
